# Oral fenofibrate for hyperbilirubinemia in term neonates: A single-blind randomized controlled trial

**DOI:** 10.1017/cts.2023.35

**Published:** 2023-03-10

**Authors:** Seyed Hossein Saadat, Rakhshaneh Goodarzi, Babak Gharaei

**Affiliations:** 1 Department of Neonatology, Clinical Research Development Center of Children’s Hospital, Hormozgan University of Medical Sciences, Bandar Abbas, Iran; 2 Student Research Committee, Faculty of Medicine, Hormozgan University of Medical Sciences, Bandar Abbas, Iran

**Keywords:** Fenofibrate, neonatal jaundice, bilirubin

## Abstract

**Background:**

Hyperbilirubinemia is common in the neonatal period; however, delayed diagnosis or inadequate treatment can cause irreparable damage to the neonates. We aimed to evaluate the efficacy of oral fenofibrate for hyperbilirubinemia in term neonates.

**Methods:**

This single-blind randomized controlled trial included 86 term neonates aged 3–7 days, with birth weight ≥2500 g, admitted to Bandar Abbas Children’s Hospital, Bandar Abbas Iran, from July 23, 2019, to July 22, 2020. The fenofibrate group received 10 mg/kg oral fenofibrate and phototherapy, while controls only received phototherapy. Serum total bilirubin was measured at 24 and 48 h and at the time of discharge. Hospital length of stay was also noted.

**Results:**

The two study groups were comparable regarding age, gender, gestational age, birth weight, and baseline total serum bilirubin levels. Serum total bilirubin levels at 48 h (*P* < 0.001) and at discharge (*P* < 0.001) were significantly lower in the fenofibrate group compared to controls. Although hospital length of stay was lower in the fenofibrate group compared to controls, the difference was not statistically significant (*P* = 0.612). Fenofibrate was more effective on the reduction of serum bilirubin in neonates aged 3–4.5 days starting at the 24^th^ hour. Moreover, it was more effective in female neonates compared to males starting at the 48^th^ hour.

**Conclusions:**

A single dose of oral fenofibrate reduced total serum bilirubin in term neonates with hyperbilirubinemia without any side effects; however, this effect was more prominent after 48 h.

## Introduction

Hyperbilirubinemia affects 60% of term neonates in their first week of life [1]. Neonatal jaundice can be a leading cause of morbidity if treated improperly [2]. It can also lead to serious complications such as temporal and occipital lobe seizures in its severe forms [3], as well as kernicterus and potentially permanent neuro-developmental delay [4].

Phototherapy is the mainstay of treatment for unconjugated hyperbilirubinemia, due to its simplicity, inexpensiveness, noninvasiveness, and easy applicability [5]. Although conventional phototherapy is widely used, some neonates still may require exchange transfusion or intensive phototherapy [6]. Also, interruption of breastfeeding and maternal-neonatal bond can be some disadvantages of phototherapy [7].

Fenofibrate and clofibrate belong to a class of drugs known as fibrates, which are phenoxyisobutyric acid derivatives, primarily administered in adult hyperlipidemia [8]. They can activate peroxisome proliferator-activated receptor alpha (PPAR-α) and thereby exert their hypolipidemic effect. The same mechanism has been shown to reduce bile acid synthesis in rats [9]. While fenofibrate’s action is similar to clofibrate, it has a better safety profile; thus, it can be safer in children compared to clofibrate [10]. Moreover, fenofibrate appears to affect bilirubin metabolism by inducing conjugation and excretion of bilirubin [10]. Minimal side effects have been observed with the prolonged use of fenofibrate for adult hyperlipidemia [11]. Furthermore, the limited studies investigating the efficacy of fenofibrate for the treatment of neonatal hyperbilirubinemia have shown promising results [10,12]; however, due to some limitations of these studies, such as small sample size, lack of randomization, inappropriate outcome assessment, and heterogeneity of the study groups, further studies are required in this regard. Also, most of previous studies had evaluated clofibrate in the class of fibrates. Here, we aimed to compare the efficacy of oral fenofibrate plus phototherapy with phototherapy alone for hyperbilirubinemia in term neonates.

## Methods

### Participants

This single-blind randomized controlled trial included term neonates referred to Bandar Abbas Children’s Hospital, Bandar Abbas, Iran, for phototherapy, from July 23, 2019, to July 22, 2020. Inclusion criteria were gestational age ≥37 weeks, birth weight ≥2500 g, age ≥72 h and ≤7 days, and total serum bilirubin ≥15 mg/dL and ≤20 mg/dL. Exclusion criteria were hemolytic disorders, such as ABO and Rh incompatibility, direct hyperbilirubinemia >2 mg/dL, glucose-6-phosphate dehydrogenase (G6PD) deficiency, systemic diseases and infection, cephalohematoma, bowel obstruction, asphyxia, congenital anomalies or chromosomal abnormalities, and maternal use of phenobarbital before birth or its neonatal use after birth. The sample size was calculated as at least 35 patients in each group using data from the study by Dabour et al.[13], with mean bilirubin level of 12.36 ± 3.98 mg/dL in the intervention group and 15.59 ± 2.39 mg/dL in the control group, α = 0.01, and power of 90%.

### Study Design

**Fig. 1.**
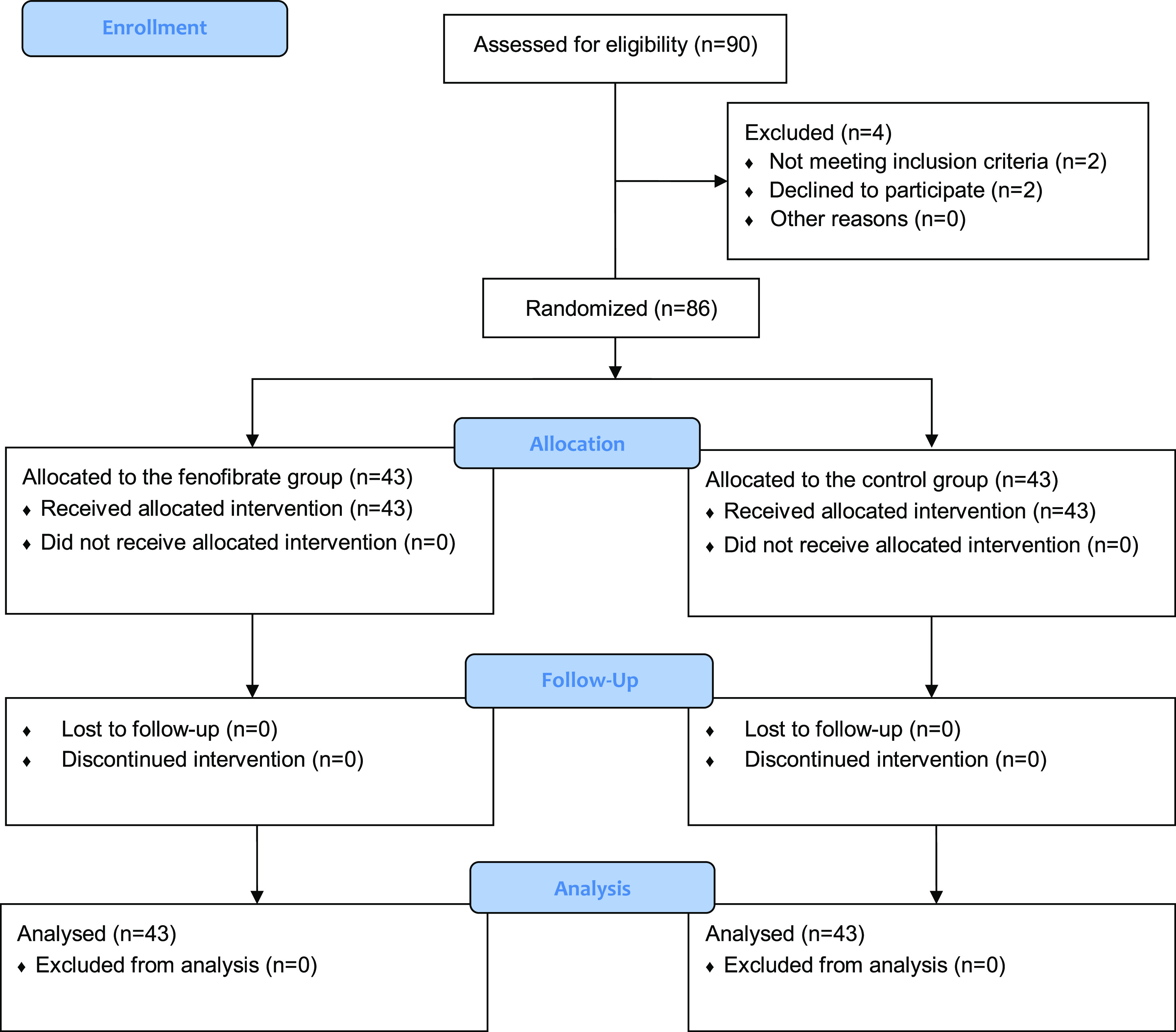
Details of patient enrollment, group allocation, and analysis.

Initially, 90 patients were evaluated for eligibility to enter the study. Of these, 2 were excluded and parents of 2 did not consent to participate. Details of patient enrollment, allocation, and analysis are shown in Fig. 1. The remaining 86 were randomly allocated to two groups using the simple randomization method and randomly generated numbers using the Random Allocation software. First, patients’ age, gender, gestational age, and birth weight were recorded. Patients of both groups received phototherapy according to the American Academy of Pediatrics protocols [6], using identical devices (Tosan Co., Iran). Patients in the fenofibrate group also received 10 mg/kg oral fenofibrate (Sobhan Darou Co., Iran) by dissolving 100 mg fenofibrate in 5 ml of distilled water (with fenofibrate concentration of 20 mg/mL). Total serum bilirubin was measured in random blood samples collected from all the patients before the initiation of treatment and then at 24 and 48 h and at discharge. Bilirubin measurement was done using the caffeine reagent and direct spectrophotometry. The personnel in charge of laboratory evaluations were blinded to the patient groupings. Drug reactions were also evaluated during the study period.

### Data Analysis

We used the Statistical Package for the Social Sciences (SPSS) software (version 25.0, Armonk, NY: IBM Corp., USA) for data analysis. The continuous variables were described using means and standard deviations. The categorical variables were described using frequencies and percentages.

Fisher’s exact test was used to compare categorical data. For the comparison of bilirubin levels between the two groups, since the sample size was over 30 in each group and based on the central limit theorem [14], the independent t-test was applied. The distribution normality of continuous variables was tested using the Kolmogorov-Smirnov normality test for subgroup analysis. Accordingly, the independent t-test was used to compare normally distributed variables and the Mann-Whitney test for variables without normal distribution. The Friedman test was used to compare bilirubin levels in each group at different time points. P-value <0.05 was considered statistically significant.

## Results

From the 86 neonates included in this study with a mean age of 4.85 ± 1.27 days, 38 (44.2%) were male and 48 (55.8%) were female. Patients in both groups were comparable regarding age, gender, gestational age, birth weight, and baseline total serum bilirubin levels (Table 1).

**Table 1.** Comparison of baseline characteristics between groups

Variables	Total (n = 86)	Fenofibrate (n = 43)	Control (n = 43)	P-value*
Age (days) mean ± SD	4.58 ± 1.27	4.63 ± 1.23	4.54 ± 1.31	0.536
Gender N (%)				
Male	38 (44.2)	22 (51.2)	16 (37.2)	0.193^†^
Female	48 (55.8)	21 (48.8)	27 (62.8)	
GA (weeks) mean ± SD	38.20 ± 1.12	38.19 ± 0.98	38.21 ± 1.25	0.759
Birth weight (g) mean ± SD	3017.09 ± 377.38	3052.79 ± 427.98	2981.40 ± 320.08	0.384^‡^
Serum total bilirubin (mg/dL) mean ± SD	17.46 ± 1.62	17.31 ± 1.56	17.61 ± 1.67	0.368^‡^

Abbreviations: N, number; SD, standard deviation; GA, gestational age.

* Analyzed by Mann-Whitney test.

† Analyzed by chi-square test.

‡ Analyzed by independent t-test.

Serum total bilirubin did not differ significantly between groups at 24 h; however, bilirubin levels were significantly lower in the fenofibrate group at 48 h and at discharge (*P* < 0.001) (Table 2).

**Table 2.** Comparison of serum total bilirubin levels between groups at different time points

Serum total bilirubin (mg/dL)*	Fenofibrate (n = 43)	Control (n = 43)	P-value^†^
Baseline	17.31 ± 1.56	17.61 ± 1.67	0.383
At 24 h	11.69 ± 2.35	12.63 ± 2.28	0.064
At 48 h	9.02^‡^ 1.16	10.16 ± 1.61	<0.001
At discharge	8.81 ± 0.97	9.75 ± 1.06	<0.001
P-value^‡^	<0.001	<0.001	

Abbreviations: N, number.

* Expressed in mean ± standard deviation (SD).

† Analyzed by the independent t-test.

‡ Analyzed by Friedman test.

**Fig. 2.**
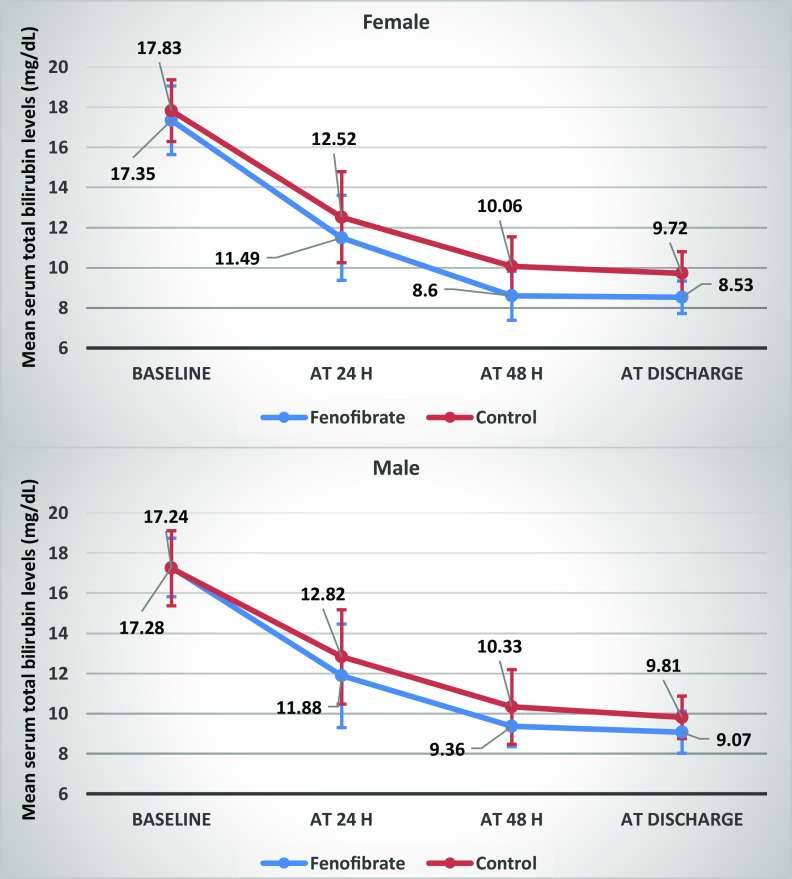
Comparison of serum total bilirubin levels between groups at different time points by gender. Error bars indicate standard deviation.

When patients were divided by gender, total bilirubin levels did not differ between groups at 24 h in both genders. Also, in male patients, the difference between groups was not significant at 48 h; however, in female patients, bilirubin levels were significantly lower in the fenofibrate group compared to controls at this time point (*P* = 0.004). Moreover, there was a significant difference in serum total bilirubin levels between groups in both male and female patients at discharge (Table 3, Fig. 2).

**Table 3.** Comparison of serum total bilirubin levels between groups at different time points by gender

Gender	Serum total bilirubin (mg/dL)*	Fenofibrate (n = 43)	Control (n = 43)	P-value^†^
Male	Baseline	17.28 ± 1.46	17.24 ± 1.87	0.944
	At 24 h	11.88 ± 2.58	12.82 ± 2.35	0.257
	At 48 h	9.36 ± 1.01	10.33 ± 1.86	0.002
	At discharge	9.07 ± 1.05	9.81 ± 1.06	<0.001
	P-value§	<0.001	<0.001	
Female	Baseline	17.35 ± 1.71	17.83 ± 1.54	0.318^‡^
	At 24 h	11.49 ± 2.12	12.52 ± 2.27	0.120
	At 48 h	8.60 ± 1.22	10.06 ± 1.48	0.004
	At discharge	8.53 ± 0.81	9.72 ± 1.08	<0.001
	P-value§	<0.001	<0.001	

Abbreviations: N, number.

* Expressed in mean ± standard deviation (SD).

† Analyzed by Mann-Whitney test.

‡ Analyzed by independent t-test.

§ Analyzed by Friedman test.

**Fig. 3.**
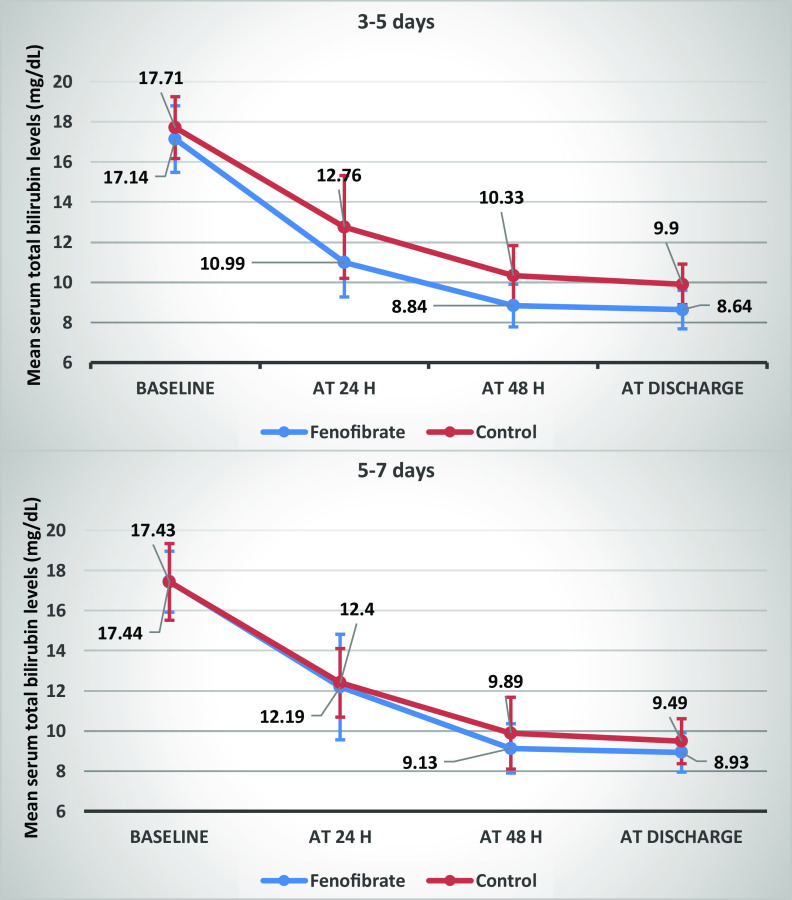
Comparison of serum total bilirubin levels between groups at different time points by age. Error bars indicate standard deviation.

Further, patients in both groups were divided by age; therefore, 27 patients in the control group and 18 in the fenofibrate group had 3–4.5 days of age, while 16 in the control group and 25 in the fenofibrate group had 5–7 days of age. The difference regarding serum bilirubin was not significant at any time points in patients aged 5–7 days. Nonetheless, at 24 and 48 h and at discharge, total bilirubin was significantly lower in the fenofibrate group compared to controls in patients aged 3–5 days (Table 4, Fig. 3).

**Table 4.** Comparison of serum total bilirubin levels between groups at different time points by age

Age	Serum total bilirubin (mg/dL)*	Fenofibrate (n = 43)	Control (n = 43)	P-value^†^
3–5 days	Baseline	17.14 ± 1.66	17.71 ± 1.54	0.251
	At 24 h	10.99 ± 1.72	12.76 ± 2.56	0.014
	At 48 h	8.84 ± 1.06	10.33 ± 1.50	0.004^‡^
	At discharge	8.64 ± 0.96	9.90 ± 1.01	0.001^‡^
	P-value§	<0.001	<0.001	
5–7 days	Baseline	17.44 ± 1.52	17.43 ± 1.91	0.993^‡^
	At 24 h	12.19 ± 2.63	12.40 ± 1.71	0.783^‡^
	At 48 h	9.13 ± 1.23	9.89 ± 1.79	0.267^‡^
	At discharge	8.93 ± 0.98	9.49 ± 1.12	0.099^‡^
	P-value§	<0.001	<0.001	

Abbreviations: N, number.

* Expressed in mean ± standard deviation (SD).

† Analyzed by Mann-Whitney test.

‡ Analyzed by independent t-test.

§ Analyzed by Friedman test.

Length of hospital stay was slightly lower in the fenofibrate group compared to the control group; however, the difference was not statistically significant. Similar results were found when patients were divided by age and gender. On the other hand, duration of phototherapy was significantly shorter in the fenofibrate group. This was also true when patients were divided by age and gender (Table 5). No drug reactions were reported in the fenofibrate group.

**Table 5.** Comparison of length of hospital stay and duration of phototherapy between groups

Variable	Fenofibrate (n = 43)	Control (n = 43)	P-value*
Length of hospital stay (days) mean ± SD	2.84 ± 0.57	2.93 ± 0.77	0.612
Male	2.95 ± 0.58	3.06 ± 0.85	0.827
Female	2.71 ± 0.56	2.85 ± 0.72	0.423
3–5 days of age	2.72 ± 0.58	2.93 ± 0.78	0.466
5–7 days of age	2.92 ± 0.57	2.94 ± 0.77	0.781
Duration of phototherapy (hours) mean ± SD	62.09 ± 13.79	76.33 ± 18.44	<0.001
Male	64.91 ± 13.81	79.50 ± 20.49	0.017
Female	59.14 ± 13.46	74.44 ± 17.23	<0.001
3–5 days of age	59.33 ± 13.79	76.22 ± 18.74	0.001
5–7 days of age	64.08 ± 13.72	76.50 ± 18.53	0.004

Abbreviations: N, number; SD, standard deviation.

* Analyzed by Mann-Whitney test.

## Discussion

In the current study, we found significantly lower total bilirubin levels with fenofibrate and phototherapy, 48 h after initiation of treatment, compared to phototherapy alone. Also, bilirubin levels were significantly lower in the fenofibrate group at the time of discharge. However, the difference between groups regarding bilirubin levels was not statistically significant at 24 h. Moreover, duration of phototherapy was significantly shorter with fenofibrate in our study. On the contrary, the results of a recent study by Prabha and Saravanan found no significant difference in bilirubin levels between the fenofibrate plus phototherapy and the phototherapy alone groups after 48 h [15]. They evaluated the duration of phototherapy as well and reported comparable values in both groups [15]. Zamiri-Miandoab et al. reported similar findings in their systematic review and meta-analysis [16]. However, Pathak et al. demonstrated significantly lower bilirubin levels with fenofibrate at 24 and 48 h of starting phototherapy. Duration of phototherapy was also significantly lower with fenofibrate in their study [17]. Gohil et al. reported similar findings [18]. Interestingly, Awad et al. compared fenofibrate for 1 day or 2 days with placebo and showed significant reduction of bilirubin levels at 36, 48, and 72 h from intervention with either fenofibrate groups compared to placebo [19]; nonetheless, there was no difference between the two fenofibrate groups in this regard, showing that a single dose of fenofibrate is as effective as its double dosing. Another study with findings inconsistent with ours was the case-control study conducted by Gowda et al., who reported comparable decline in bilirubin levels with fenofibrate and phototherapy compared to phototherapy alone [20]; nevertheless, they used a 5 mg/kg dose of fenofibrate, showing that this dose is not sufficient for the induction of glucuronyl transferase enzyme, a major contributor to bilirubin metabolism. Other studies in the literature have all shown the effectiveness of fenofibrate on the reduction of neonatal bilirubin levels, while these effects started at different times after the intervention [10,12,21,22]. The heterogeneity of findings across these studies can result from the difference in inclusion and exclusion criteria, demographic and other general characteristics of populations under study, the accuracy and the method of bilirubin measurements, the device and protocol used for phototherapy, and the timing of bilirubin measurements.

With regard to hospital length of stay, we found shorter duration of hospitalization with fenofibrate compared to controls; however, the difference did not reach statistically significant levels. On the contrary, duration of hospital stay was significantly lower with fenofibrate in Pathak et al.’s study [17]. Awad et al. also demonstrated significantly shorter hospital stay with fenofibrate [19]. This discrepancy can be justified by the difference in the phototherapy protocol, the efficiency of phototherapy devices, and the overall quality of patient care in the two studies. Lower baseline serum bilirubin levels in Pathak et al.’s study can be taken into consideration as well. Furthermore, by reviewing the baseline bilirubin levels of the 3 groups in Awad et al.’s study, we found that at least a number of patients had values higher than 20 mg/dL; these patients had been excluded from our study. Moreover, they included both hemolytic and nonhemolytic cases of neonatal jaundice [19]. Reduction of hospital stay with fenofibrate has also been reported in some other studies [10,21–23].

As for drug reactions and side effects, none were observed in the current study. This was in line with the findings of Pathak et al. [17]. Similarly, no side effects were reported for fenofibrate in a recent meta-analysis of 6 studies [16], indicating that fenofibrate can safely be used for neonatal hyperbilirubinemia. This has been confirmed by other studies [10,18,21,22].

The major strength of the current study was that patients in both groups were homogenous with regard to potential effectors or confounding variables. In addition, we excluded patients with hemolytic disorders, which was not the case in many previous studies. Moreover, we further divided patients by gender and age and found interesting results: fenofibrate was more effective on the reduction of serum bilirubin in neonates aged 3–5 days starting at the 24^th^ hour. Also, it was more effective in female neonates compared to males starting at the 48^th^ hour.

Our study was not without limitations. Duration of phototherapy had been evaluated in some studies as an indicator of fenofibrate efficacy, which we did not record. Another limitation was the small sample size that could limit the generalizability of the results.

## Conclusions

The results of the current study confirmed the efficacy of fenofibrate as an adjunct to phototherapy for the treatment of neonatal hyperbilirubinemia. A single dose of oral fenofibrate reduced total serum bilirubin in term neonates with hyperbilirubinemia without any side effects; however, this effect was more prominent after 48 h. What was further found in the current study was that females and younger neonates benefited more from the effects of fenofibrate.

## Data Availability

The datasets used and/or analyzed during the current study are available from the corresponding author on reasonable request.

## Trial Registration

Iranian Registry of Clinical Trials (IRCT), IRCT20201209049660N1. Registered 24/12/2020. Retrospectively registered, https://www.irct.ir/user/trial/52901.
